# Towards interpretable machine learning models for diagnosis aid: A case study on attention deficit/hyperactivity disorder

**DOI:** 10.1371/journal.pone.0215720

**Published:** 2019-04-25

**Authors:** Sarah Itani, Mandy Rossignol, Fabian Lecron, Philippe Fortemps

**Affiliations:** 1 Fund for Scientific Research - FNRS (F.R.S.- FNRS), Brussels, Belgium; 2 Department of Mathematics and Operations Research, Faculty of Engineering, University of Mons, Mons, Belgium; 3 Department of Cognitive Psychology and Neuropsychology, Faculty of Psychology and Education, University of Mons, Mons, Belgium; 4 Department of Engineering Innovation Management, Faculty of Engineering, University of Mons, Mons, Belgium; University of Craiova, ROMANIA

## Abstract

Attention Deficit/Hyperactivity Disorder (ADHD) is a neurodevelopmental disorder that has heavy consequences on a child’s wellbeing, especially in the academic, psychological and relational planes. The current evaluation of the disorder is supported by clinical assessment and written tests. A definitive diagnosis is usually made based on the DSM-V criteria. There is a lot of ongoing research on ADHD, in order to determine the neurophysiological basis of the disorder and to reach a more objective diagnosis. The advent of Machine Learning (ML) opens up promising prospects for the development of systems able to predict a diagnosis from phenotypic and neuroimaging data. This was the reason why the ADHD-200 contest was launched a few years ago. Based on the publicly available ADHD-200 collection, participants were challenged to predict ADHD with the best possible predictive accuracy. In the present work, we propose instead a ML methodology which primarily places importance on the explanatory power of a model. Such an approach is intended to achieve a fair trade-off between the needs of performance and interpretability expected from medical diagnosis aid systems. We applied our methodology on a data sample extracted from the ADHD-200 collection, through the development of decision trees which are valued for their readability. Our analysis indicates the relevance of the limbic system for the diagnosis of the disorder. Moreover, while providing explanations that make sense, the resulting decision tree performs favorably given the recent results reported in the literature.

## Introduction

Attention Deficit/Hyperactivity Disorder (ADHD) is a neuropsychiatric disorder which has an estimated overall prevalence of five to seven percent of youngsters [1]. Despite the neurocognitive origins of the syndrome, the clinical diagnosis of ADHD mainly relies on behavioral symptoms of inattention, hyperactivity and/or impulsivity, persisting for at least 6 months; such symptoms occuring before the age 12 and leading to the impairment of familial, social, or academic functioning [2]. More than ten years ago, it was claimed that the criteria established by the Diagnostic and Statistical Manual of Mental Disorders (DSM) are necessary but not sufficient for ADHD diagnosis [3]; there is still a need for more objective criteria on that regard. Yet, neuroimaging studies showed consistent structural and functional neural alterations related to ADHD [4, 5]. In order to provide objective observations, such alterations may be considered to complete the current assessment of the disorder and accordingly, to increase the agreement between clinicians, which is currently estimated at 61.0% [6].

In early Magnetic Resonance Imaging (MRI) studies [7], the comparison of neuroanatomical data on control subjects and ADHD subjects showed that there were decreased volumes around the prefrontal-striatal system of the ADHD subjects’ brains. Later studies extended this observation to other brain regions, including the anterior cingulate cortex, the frontal cortex [8, 9]; and the ventral striatum included in the reward circuit [10]. Decreased cortical thickness was also found in the right hemisphere [11]; in localized areas such as parietal and motor zones [10]; in areas located in the attentional circuit [12]; and more generally in the brain [13, 14]. Functional Magnetic Resonance Imaging (fMRI) has also been widely involved in the characterization of brain activity in ADHD. The current trend calls for the assessment of the brain connectivity, rather than focusing on isolated area dysfunctions [15]. Resting-state and task-based studies showed dysfunctions in several networks such as the default mode, affective and attention ones [16, 17]. Depending on the predominant patterns in ADHD subjects (e.g. attention deficit, impulsivity), some can exhibit hypo-activity in frontoparietal networks or hyper-activity in frontal-striatal-cerebellar connections [15]. Other studies showed isolated dysfunctions, e.g. in the amygdala during emotion processing [18].

Despite the considerable number of studies related to ADHD, the disorder remains subject to the absence of a common etiology. In this regard, the advent of Machine Learning (ML) is expected to provide new insights. As suggested by [19], ML methods differ from standard statistical ones in some respects. On the one hand, ML is perceived as a promising alternative way of conducting exploratory data analyses which are inductive and assumption-free [20–22]. Indeed, ML methods are implemented to extract general patterns and relations from observational data [19]. On the other hand, statistical analyses are hypothetico-deductive in nature, which means that experimental data should be collected to test initial assumptions [22]. Such an approach may impede the compilation of large datasets and simultaneously, the acquisition of results with a high level of confidence. Moreover, while it would be interesting to obtain a more comprehensive description of neuropathologies, statistical analyses allow to test assumptions on isolated functional and/or structural characteristics of these neuropathologies. Finally, as analysis tools, statistical approaches are less adapted to build assessment models with a diagnosis perspective. Through a dual potential of knowledge inference and prediction, ML has thus attracted growing interest in the sphere of translational neuroscience over the last years, in the hopes of solving questions which currently remain pending—including the etiological basis of ADHD.

Attention deficit/hyperactivity disorder has been significantly targeted by ML studies. To this day, Support Vector Machines (SVM) have been the most considered predictive models [23–31]. More recently, deep learning has also been given consideration [32, 33]. Admittedly, such models provide satisfaction on prediction accuracy, but the related predictions may be hardly interpreted. Yet, for the purpose of diagnosis aid, the interpretability of a model is a quality that is imperative to reach, since it ensures that (1) the model is able to infer a patient’s state with comprehensive justifications; (2) the model may lead to a better understanding of the disorder [20, 21, 34, 35]. Actually, such a goal is in line with the recent paradigm of Theory-Guided Data Science (TGDS) [36]. Based on the extensive use of both data and the existing scientific knowledge, TGDS is intended to achieve the development of data science models having a better practicality in different scientific fields.

Considering the foregoing, interpretability is the key to effective decision aid. Powerful tools are found in the literature for the purpose of interpretable modeling. In this respect, *observations* derived from measuring inputs (e.g. fMRI voxels) are processed to raise explainable *factors* [37]. Forward models such as Independent Component Analysis (ICA) and General Linear Model (GLM) aim to recover sources generating the observations. The Spatial Filtering Method (SFM) [38] is another example of analysis tool which was specifically developed to perform the linear transform of fMRI timeseries into discriminative signals [38, 39]. Though all these methods perform data transform, it remains possible to raise the influence of the original data through the analysis of the transform matrix which maps the observations and the factors [37]. However, it was shown that discriminative features may be found without the need of transforming data, which makes interpretability simpler. For instance, it is possible to extract some basic explanatory features from neuroimaging data, such as the variance of fMRI timeseries, to perform the effective classification of neurotypical and ADHD subjects [40]. In this case, it remains important that such features are presented as input variables of interpretable classifiers in order to understand the underlying decision mechanisms. Decision trees are amongst the most well-known interpretable classifiers, and are recognized as particularly attractive for diagnosis aid processes [41]. Indeed, any prediction made by a decision tree can be justified through a decision chain (with causal relations), including only the most discriminative and interpretable (since they are not transformed) features that resulted in the final decision.

In the present work, we propose a TGDS approach in the context of medical diagnosis, with the overall objective of developing predictive models which heed the knowledge of their final user. Such a purpose is achieved through the interaction with the user, i.e. the medical expert, throughout the process. The method starts with the automatic development of a first predictive model. In a second stage, an expert assesses the need of revising the model, and suggests some avenues for improving it, if required. We use decision trees to illustrate this stepwise approach. The contributions of our work are exposed below.

- We advocate for a ML methodology which focuses on the explanatory power of a model instead of its lonely predictive accuracy. For such a purpose, we adopted an expert-aware approach [20] with the aim of increasing the final users’ (i.e. the clinicians) trust on ML models. We show that readable models such as decision trees are well-suited to conduct such an approach. Indeed, the readability of a model allows us to understand how a decision is made, and to assess the extent to which the related explanations are consistent.
- We propose new and interesting results regarding the data used in this study: the ADHD-200 collection. A significant part of the ML research on ADHD was derived from this dataset, which was released at the occasion of a contest in 2012 [40, 42, 43]. Since then, research has been ongoing to better understand and predict ADHD: our study is also working towards this objective. In comparison to the recent literature on predictive accuracy, our results were favorable.
- Our work shows that it is possible, through ML, to confirm previous findings of the neuroscience literature, based on larger datasets. In particular, our results suggest that ADHD has some relation with the limbic system, which gives prospect for thorough consideration in the sphere of neuroscientific research.

## Materials and methods

In our work, we considered a data sample extracted from the open and freely available ADHD-200 collection [42]. We present the data in the first part of the section. Then, we explain the use of decision trees as predictive models. Finally, we reveal the analysis methodology of our study.

### Data

#### Overview on the ADHD-200 competition

The data used in our study were released in the context of the ADHD-200 competition (2012) [42–45]. The international contest challenged research teams to propose a model that would be able to predict ADHD with the best possible accuracy. The ADHD-200 collection, a large compilation of clinical and imaging data, was proposed for such a purpose. This open dataset results from a collaborative work of eight imaging sites based in China, the Netherlands and the United States. Though the contest ended, research has been ongoing in the objective of better understanding ADHD and improving the prediction accuracy of 61.5%—an accuracy that was achieved at the end of the competition [46, 47].

Upon the creation of a free NITRC—NeuroImaging Tools and Resources Collaboratory—account (www.nitrc.org), the ADHD-200 consortium gives full, unrestricted access to the ADHD-200 collection. For each site, the ADHD-200 collection provides training and test sets including Typically Developing (TD) and ADHD subjects. The *training set* is used to develop predictive models and the *test set* is kept separately for validation. Different strategies were considered to deal with such a multi-site dataset [40]. One possibility is to merge all the training sets into the same dataset and to train a single predictive model. Given the heterogeneity of the multi-site dataset, another strategy consists of training several models, based on homogeneous subsets extracted from the collection. Finally, outside the context of the ADHD-200 competition, one could be interested in studying a given population, thus selecting the data of a single site.

The segmentation into training and test sets was imposed by the ADHD-200 collection itself. During the competition, only the training sets were made available to the competitors. The predictive models that were proposed in the competition were later assessed against test sets which were made available by the ADHD-200 consortium sometime after the competition. Since then, most of the research works based on the ADHD-200 collection use the training and test sets as they are proposed by the ADHD-200 collection. This segmentation, kept as it is, allows the comparison with the existing literature to be easier and fairer.

#### Subjects

Our study is based on the data subset collected by the New-York University Child Study Center; we denote it as the NYU sample.

Phenotypic features include age, gender, Intellectual Quotient (IQ), as well as handedness. The IQ was assessed using the Wechsler Abbreviated Scale of Intelligence (WASI) and the handedness using the Edinburgh scale [44, 48]. A diagnosis label is also available for each patient. Parents were asked to assess their child’s behavior with the Conners Parent Rating Scale-Revised, Long version (CPRS-R: LV) instrument. Parents and children were also submitted to the Schedule of Affective Disorders and Schizophrenia for Children—Present and Lifetime Version (KSADS-PL). The inclusion criteria for Typically Developing (TD) and ADHD subjects are based on both KSADS-PL and CPRS-R: LV:

- **ADHD**—diagnosis based on KSADS-PL and T-score ≥65 with CPRS-R: LV;
- **Controls**—absence of any axis I diagnosis under KSADS-PL in parents and child and T-score <60 according to CPRS-R: LV.

General inclusion criteria involved that right-handed children with a full-scale IQ superior to 80, and not showing other persistent medical issues were recruited. For further details, the reader is referred to the website of the ADHD-200 consortium [44].

Tables 1 and 2 present the demographics of the control and ADHD groups in both the training and test sets. It should be noted that twelve subjects were excluded from the original training set (*n* = 222) because of missing phenotypic features and/or brain images.

**Table 1:** Demographics of the control group.

	Age	IQ	Gender		*n*
			F	M	
Training set	12.1 ± 3.1	110.8 ± 13.8	50	43	93
Test set	11.8 ± 3.0	114.0 ± 13.4	4	8	12

**Table 2:** Demographics of the ADHD group.

	Age	IQ	Gender		*n*
			F	M	
Training set	11.3 ± 2.7	107.0 ± 13.3	25	92	117
Test set	10.3 ± 2.5	103.3 ± 13.2	9	20	29

While the NYU training set is quite well-balanced with 55% of TD and 45% of ADHD subjects, the test set presents a different distribution (i.e. respectively 30% vs 70%). This explains why it is challenging to tackle with the NYU dataset. The development of predictive models is based on a segmentation of the training set where the TD and ADHD populations are quite well represented. An assessment of the models against a very different distribution can lead to pessimistic results. Incidentally, the results of the ADHD-200 collection were the worst on the NYU subset [47]. This is why we wanted to address this challenging subset in particular, besides our concern of studying a large and homogeneous dataset.

#### Scanning procedure & preprocessing

The ADHD-200 collection includes resting-state functional Magnetic Resonance Images (rs-fMRI) for each subject. Patients under medication were asked to drop their treatment at least twenty-four hours before image acquisition. During the fMRI run, the subjects were asked to close their eyes whilst staying awake and relawed. The functional images were acquired using Siemens Magnetom Allegra syngo MR 2004A. The parameters of acquisition are: echo time (TE) = 15 ms; repetition time (TR) = 2000 ms; flip angle (FA): 90°; voxel size = 3 × 3 × 4 mm^3^; number of slices = 33; slice thickness = 4 mm [44].

The fMRI images were preprocessed by the NeuroBureau according to the *Athena Pipeline* [45]. We detail here the main steps of this procedure, which ends with the extraction of Blood Oxygen Level-Dependent (BOLD) timeseries per voxel [43, 45]:

- first four volumes removal;
- time and motion correction;
- co-registration of the mean functional image onto the related structural image;
- writing the fMRI data into the MNI space (resolution: 4 × 4 × 4 mm^3^);
- removing the effects of physiological noise, head motion and scanner drifts;
- band-pass filtering (]0.009, 0.08[Hz) of the timeseries.

**Fig 1:**
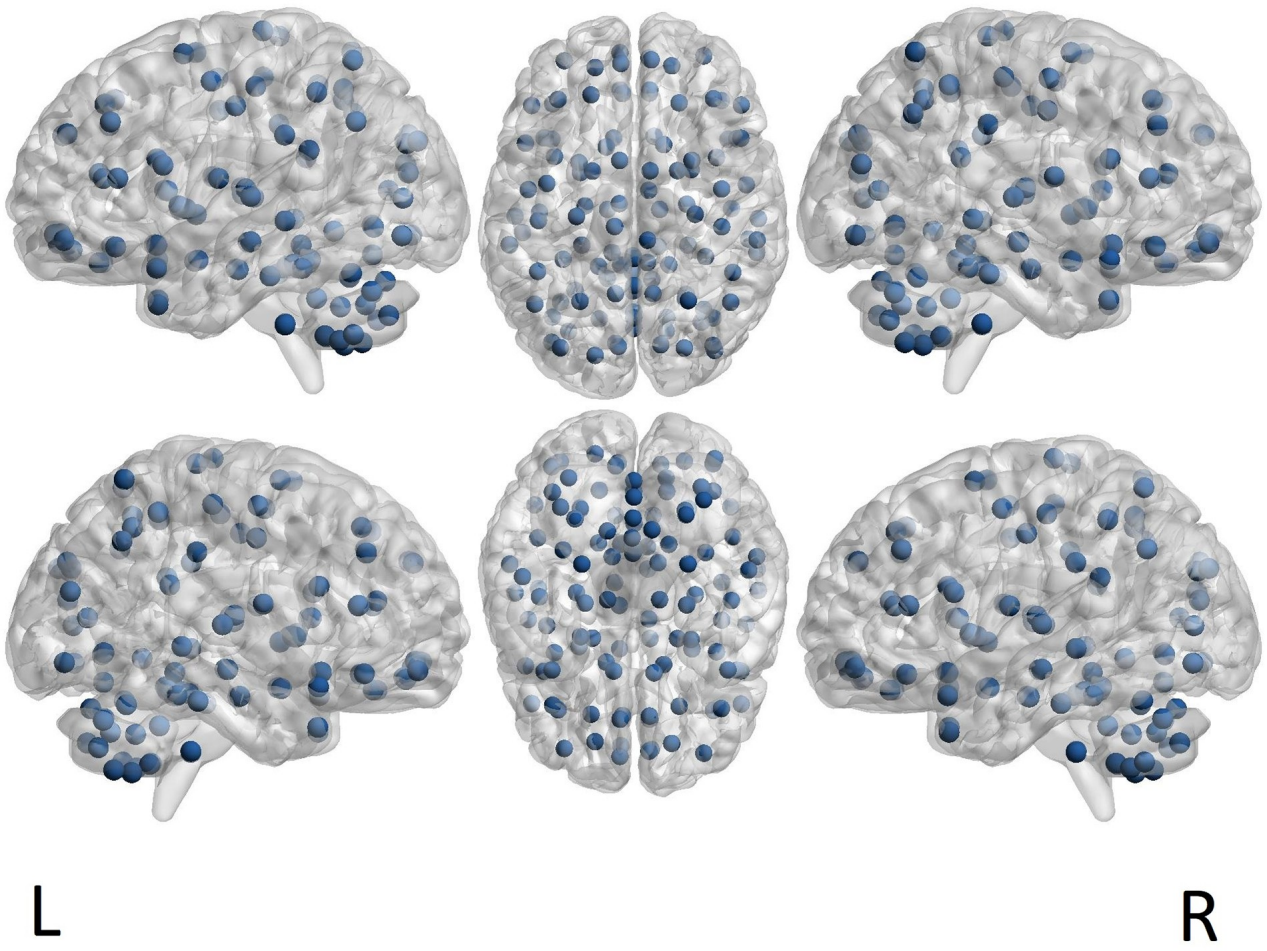
ROI centers as defined by the AAL parcellation. The brain illustration is a template provided, handled and visualized with BrainNet Viewer tool [52, 53].

A further stage of processing consists of averaging the timeseries for defined brain areas [43]. In our study, we considered the fMRI timecourses extracted for the 116 Regions of Interest (ROI) defined by the Automated Anatomical Atlas (AAL) [49] (see Fig 1). The matching between the labels of the cerebral zones and their localization in the brain is found in the Online Brain Atlas Reconciliation Tool [50]. Brain zones are numbered from 1 to 116: an even (resp. odd) number indicates a region included in the right (resp. left) hemisphere.

#### Classification features

We developed our predictive models based on both phenotypic and imaging features. Among phenotypic information, we considered the age, gender, IQ, and handedness. In addition to phenotypic information, we used the fMRI timecourses extracted per ROI according to the AAL brain parcellation. Rather than focusing on functional connectivity [51] and interactions between brain zones, we were interested in assessing the variance of each BOLD fMRI signal. Actually, our interest in such a basic information is in line with our objective of proposing interpretable models. Indeed, the variance of each signal represents a measure of energy, so each ROI is assessed individually by the dynamism of its neuronal activity. Therefore, for each patient, a set of 120 features is available for classification: 4 phenotypic attributes and 116 signal variances related to the 116 ROIs.

### Decision trees

Under an intuitive tree-based representation, decision trees are read in a top-bottom approach, in answering different questions about an instance, before making a final classification. Therefore, decision trees are transparent and intelligible models, as each prediction is justified by a decision chain, and each part of this decision chain consists of a question that may be translated literally [54]. Several variants of algorithms exist to develop decision trees [55–57]. In this work, the classifiers were computed using Weka software [58], through its J48 component. The latter achieves an implementation of algorithm C4.5 [59], on which we give a brief overview in the following paragraph.

According to the recursive and greedy C4.5 algorithm, the learning process unfolds in accordance with the logic of dividing and conquering. At the top of the tree, the root contains all the training instances which are separated on the base of a selected attribute to establish the *purest* child nodes, i.e. nodes including instances belonging for the most part even entirely, to the same class (in this case, either TD or ADHD). The transition from a level to another occurs when splitting the parent nodes based on an attribute whose pertinence for division is assessed according to the *information gain* criterion, measured by the entropy. If *t* designates a node, *N*(*t*), the number of instances included in that node (regardless the class to which they belong), *N*_*c*_(*t*), the number of instances belonging to class *c* in the same node, the entropy of the parent node is computed as [60]:

$$i\left(t\right)=-\sum_{c}\frac{N_{c}\left(t\right)}{N(t)}\log_{2}(\frac{N_{c}\left(t\right)}{N(t)})$$

it measures the impurity of the current node, i.e. its degree of heterogeneity regarding the number of classes that are represented in this node. The information gain caused by splitting the parent node *t* into two child nodes *t*_*L*_ and *t*_*R*_ is computed as:

$$\Delta i\left(t\right)=i\left(t\right)-\frac{N(t_{L})}{N(t)}i\left(t_{L}\right)-\frac{N(t_{R})}{N(t)}i\left(t_{R}\right).$$

With respect to this process, an important question should be solved about the stopping criterion. Nodes are split until the minimal number of training instances required by leaf (i.e. ending node) *m* is encountered; the parameter is defined prior to the learning process execution. Thus, the learning process is run on a set of training features which is not necessarily needed in its entirety; only the most discriminative features are used by the final model.

The parameter *m* clearly states the granularity of the models, i.e. their size. We admitted this parameter varies between 5 and 20, the latter which corresponds approximately to 10% of the size of the training set. In the range of possible values [5, 20] for parameter *m*, the one associated with the highest accuracy of the resulting model, in the sense of Leave-One-Out Cross Validation (LOOCV), was held as relevant. LOOCV does not require any partitioning into folds and is based on the assessment of models which are close to the model trained on the whole training set [57, 61]. In our opinion, these advantages make LOOCV suitable for parameter selection. Moreover, the parameter *m* expresses, in absolute value, the minimal number of instances that each leaf of the decision tree must cover. Since *m* is not a relative measure, but an absolute one, it has to be selected in a procedure involving a dataset with a similar size to the one of the whole training set. Thus, LOOCV appears here as particularly convenient to select the value of the absolute parameter *m*, while *k*-fold CV procedures would better address the tuning of relative parameters.

Let us note that the development of decision trees is a process that is highly sensitive to the content of the input training set [55]. Therefore, it is interesting to reduce the input training set, including 120 features for each patient in this case, to select the most *relevant* ones, i.e. features which are meaningful in the sense of a given criterion. Then, from this reduced set of classification features, decision trees are built based on the most *discriminative* ones, i.e. the features which contribute to make a clear distinction between the ADHD and TD subjects.

### Expert-based methodology

**Fig 2:**
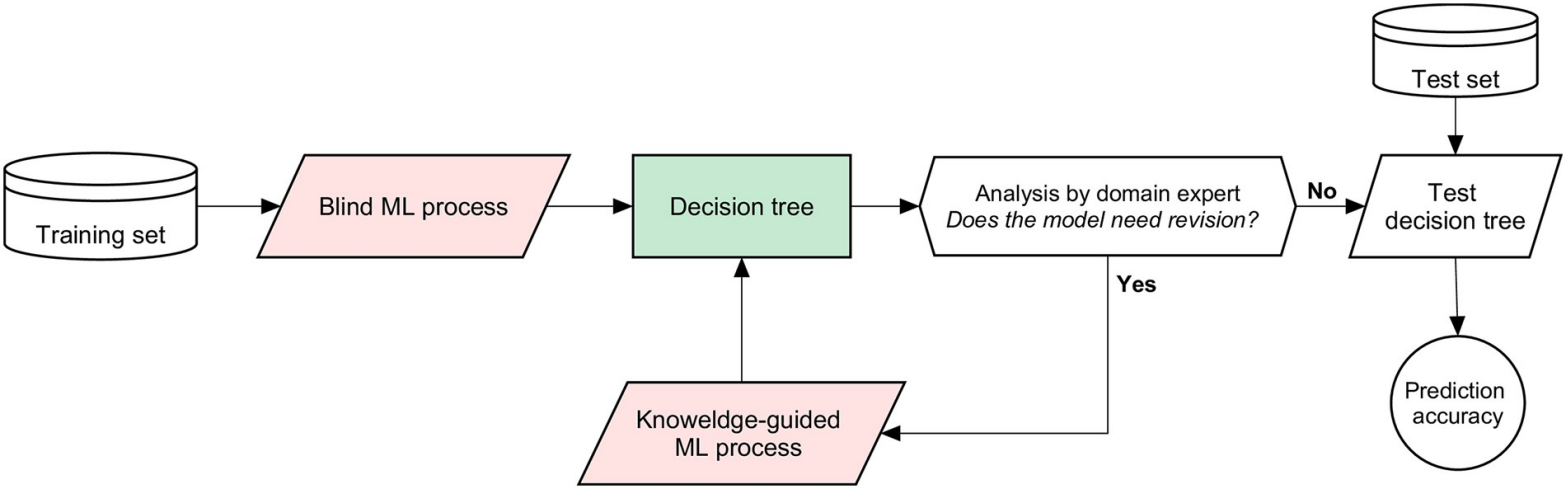
Our expert-based methodology.

**Fig 3:**
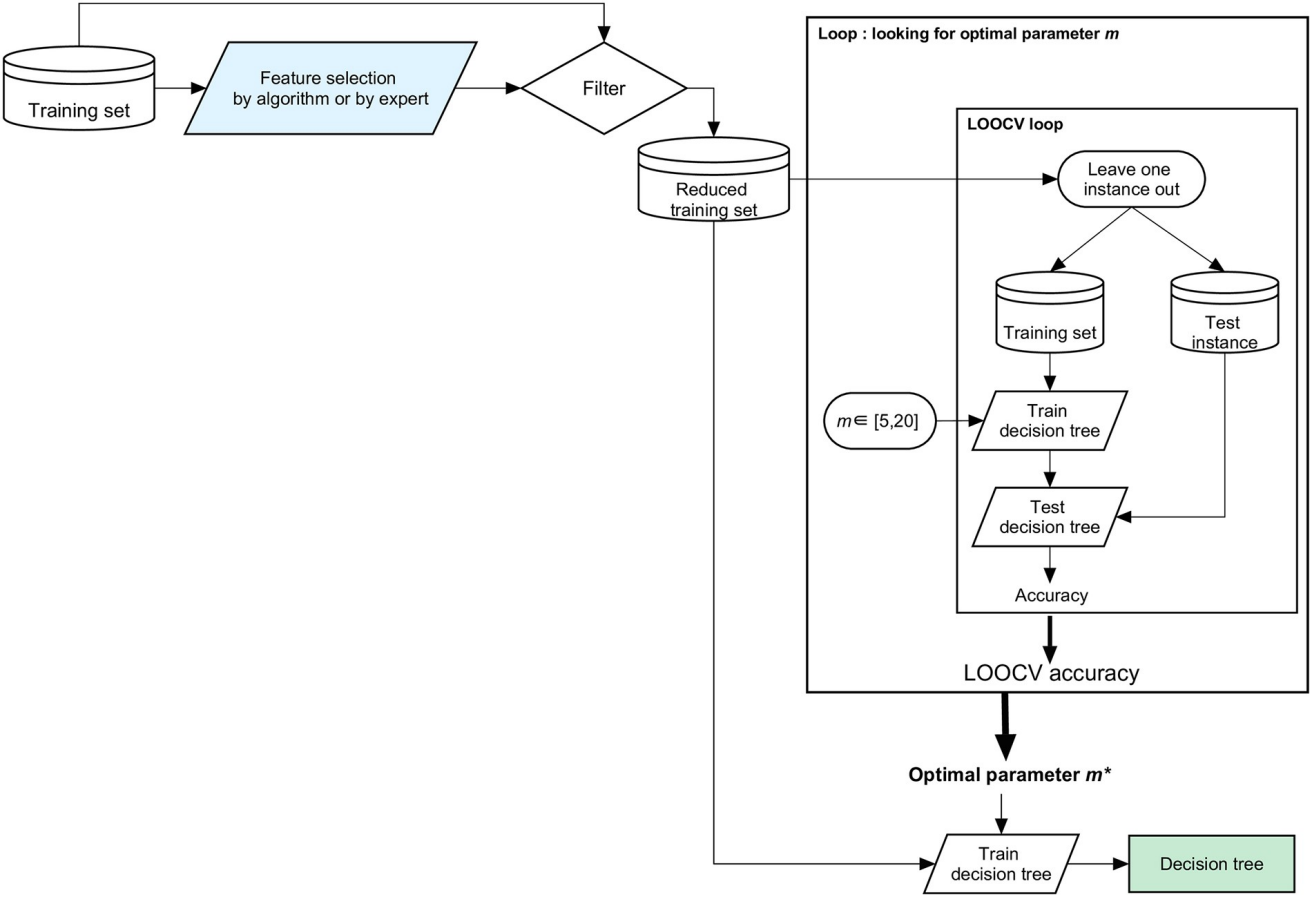
ML process.

The study was carried out in accordance with our expert-based methodology depicted in Fig 2. The principle of the approach is summarized as follows. First, a *blind ML process* (i.e. not guided by the expert) is launched: it consists of the automatic development of a predictive model. Then, an expert reads the resulting model, and assesses whether or not it makes sense. If revision is required, a *knowledge-guided ML process* is initiated with the help of the expert who provides some indications, e.g. adding, removing or extracting a subset of features, to introduce a coherent knowledge for the development of a second model. The overall methodology is thus conducted in a stepwise way to improve the quality of the prediction mechanism. Fig 3 depicts the execution of a ML process (with decision trees as classifiers). It includes feature selection and training. Note that the difference between the blind and knowledge-guided ML process lies in the selection of relevant training features. We describe these processes in more detail below.

**Fig 4:**
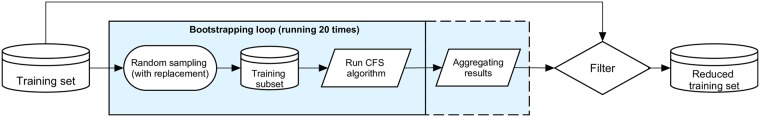
Feature selection with CFS.

- Blind ML process: this process relies on an algorithmic approach for the selection of relevant training features. In this case, we considered the Correlation-based Feature Selection (CFS, implemented by Weka) [62] which removes redundant information when extracting a subset of features that present a low inter-correlation, but that are highly correlated with the outcome variable (i.e. diagnosis in this case). This automatic method is based on the computation of Pearson’s correlations and it does not require a set correlation threshold (see [62] for further details). To ensure robust feature selection, we considered the use of an ensemble feature selection strategy [63, 64]. The procedure is presented in Fig 4 and is executed as follows.
  1. Extracting some bootstrap training samples, i.e. achieving random subsampling with replacement.
  2. Applying the selection of features on each bootstrap sample.
  3. Aggregating the results of the feature selections, given a specific rule. In this case, we selected the features which most often showed up (at least in 25% of the feature selections).
  We applied this strategy in running CFS on 20 bootstrap samples; each sample corresponds to a subset including 75% of instances extracted from the initial training set. The relevant features selected for each instance constitute the *reduced training set* (Figs 3 and 4).
- Knowledge-guided ML process: this process is driven by features suggested by the expert following the analysis of the predictive model developed previously. In this case, our expert tried to identify brain areas that are part of a reference brain system. A second decision tree was trained on the regions included in this reference system.

### Assessment

The final predictive model was assessed based on its ability to reproduce the diagnosis provided by clinicians. As shown in Fig 2, this validation was exclusively achieved on the test set which was kept separately for this purpose. We compared clinical and predicted diagnoses to measure the following parameters:

- *TP* (*TN*)—the number of true positives (negatives), i.e. pathological (healthy) patients whose diagnosis was rightly predicted;
- *FP* (*FN*)—the number of false positives (negatives), i.e. healthy (pathological) patients who were wrongly predicted as pathological (healthy).

Based on these indicators, the models proposed in this study were assessed through performance measures used in the current medical practice to evaluate clinical tests, and also considered in ML: accuracy, specificity and sensitivity rates [57, 65, 66].

- **Accuracy** measures the rate of right predictions.
  $$A=\frac{TP+TN}{TP+FP+TN+FN}=\frac{TP+TN}{\text{Nb}.\;\text{of}\;\text{subjects}}$$
- **Specificity** (true negative rate) measures the ability to detect healthy patients.
  $$tn=\frac{TN}{TN+FP}=\frac{TN}{\text{Nb}.\;\text{of}\;\text{healthy}\;\text{subjects}}$$
- **Sensitivity** (true positive rate) measures the ability to detect pathological patients.
  $$tp=\frac{TP}{TP+FN}=\frac{TP}{\text{Nb}.\;\text{of}\;\text{pathological}\;\text{subjects}}$$

We also reported the 4-fold CV accuracy (with standard deviation) of the final predictive model. This allows to assess the overall stability of the prediction rate achieved on the basis of a rough partitioning of the training data into four folds.

## Results and discussion

In this section, we give the results of our expert-based framework for the development of decision trees. As mentioned beforehand, we assess the predictive models against their explanatory power, i.e. the credibility of the decision chains. We give a summary of the results in the last part of the section.

### Blind ML process

**Fig 5:**
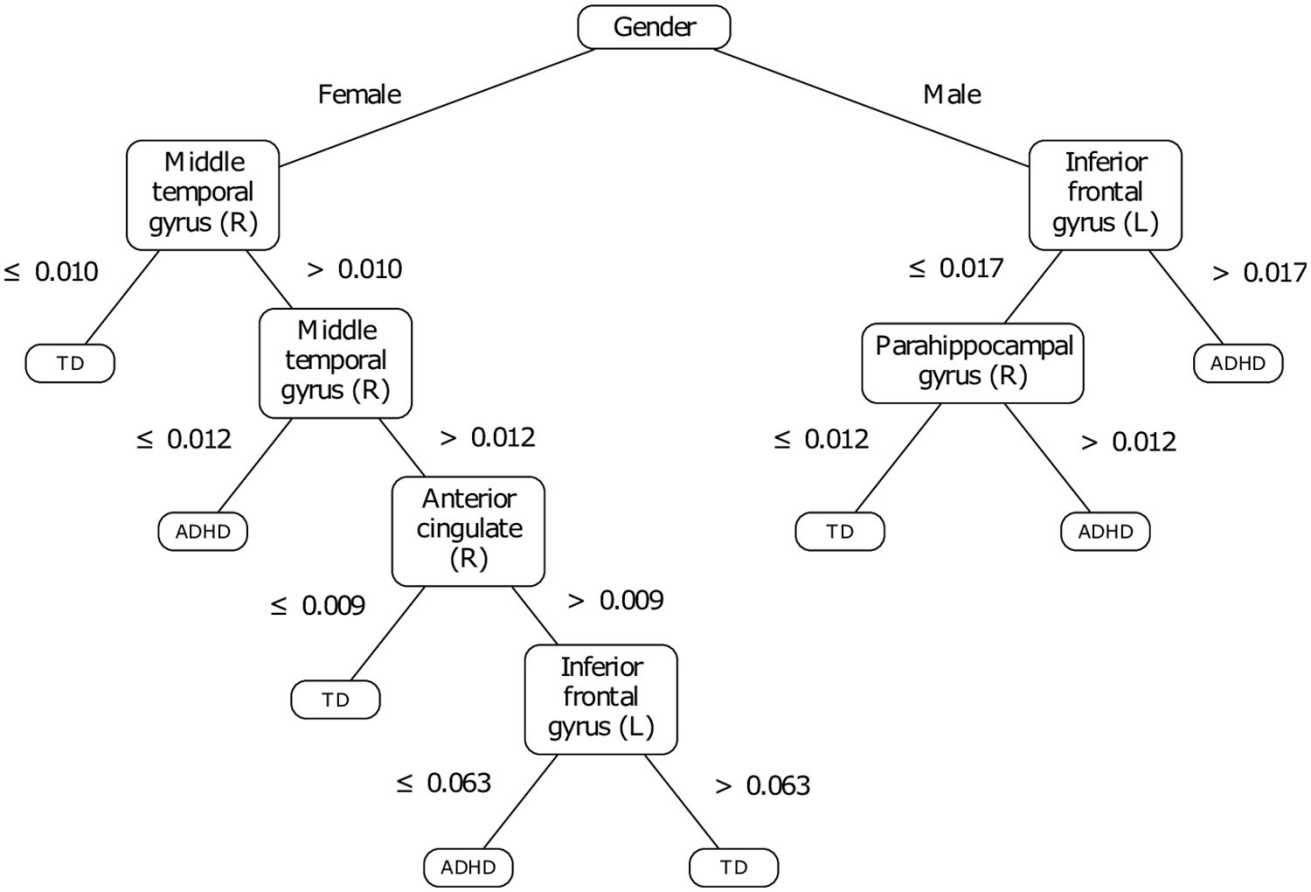
Decision tree developed without prior knowledge.

The training features remaining after the CFS reduction of dimensionality are exposed in Table 3. Let us note the regions included in the Left (resp. Right) hemisphere are denoted as L (resp. R). The decision tree that was trained based on these features is shown in Fig 5.

**Table 3:** Extracted features through automatic correlation-based selection.

Type	Attributes
Phenotype	Gender
Brain regions	15: Inferior frontal gyrus, orbital part (L)
	27: Gyrus rectus (L)
	32: Anterior cingulate and paracingulate gyri (R)
	40: Parahippocampal gyrus (R)
	70: Paracentral lobule (R)
	87: Temporal pole, middle temporal gyrus (L)
	88: Temporal pole, middle temporal gyrus (R)

First, we notice that the dimensionality reduction process selected only the gender among the phenotypic features, and a set of seven cerebral zones among the initial set of 116 ROI. Furthermore, the variance thresholds on which the tree subdivisions are based on are close to zero (see Fig 5) and represent approximately one to three percent of the maximal variance observed on the signals. In other terms, the questions raised on each cerebral zone fall under the assessment of its activation or non-activation while the patient is at resting-state. However, the question raised on the activity of the middle temporal gyrus remains difficult to interpret. Indeed, based on a ternary split, the question is not related to the (non-)activation of the zone in absolute terms: it rather brings a nuance on the intensity of such an activation.

A previous study [67] raised irregularities in ADHD children in several areas, more specifically in the temporal cortices and the right middle temporal gyrus. Interestingly, the model presented by Fig 5 confirms the involvement of the right middle temporal gyrus to make the dissociation between TD and ADHD children, but only in girls. Yet, this same structure was raised for its ability to manage cognitive processes, including interpretation and recognition tasks [68, 69]. Moreover, our model suggests that two other structures are involved in girls with ADHD: the right anterior cingulate and the left inferior frontal gyrus. Yet the right anterior cingulate was shown as involved in attention [70], by processing the selection of both the stimulus and the response; dysfunctions in ADHD subjects were reported regarding this area [71]. The left inferior frontal gyrus (IFG) was raised as critical for response inhibition: as suggested in [72], patients with damage in the left IFG perform less well a Go/NoGo task than controls, and most importantly as a higher level of inhibitory control is required.

For boys’ assessment, the decision tree suggests that the left IFG and the right parahippocampal gyrus are sufficient for diagnosis. Yet, it was demonstrated that boys with ADHD present irregularities in frontolimbic areas, which are comparable to impairments that characterize antisocial behaviors [73]. Moreover, it was shown the right parahippocampal gyrus allows the detection of infrequent events, which requires selective attention [74]. Thus, the involvement of this area in our model may indicate that ADHD-affected boys exhibit difficulty in detecting events that are relevant to them. This observation appears consistent with the findings reported in [75], suggesting impaired executive control and sustained attention amongst children with ADHD.

**Fig 6:**
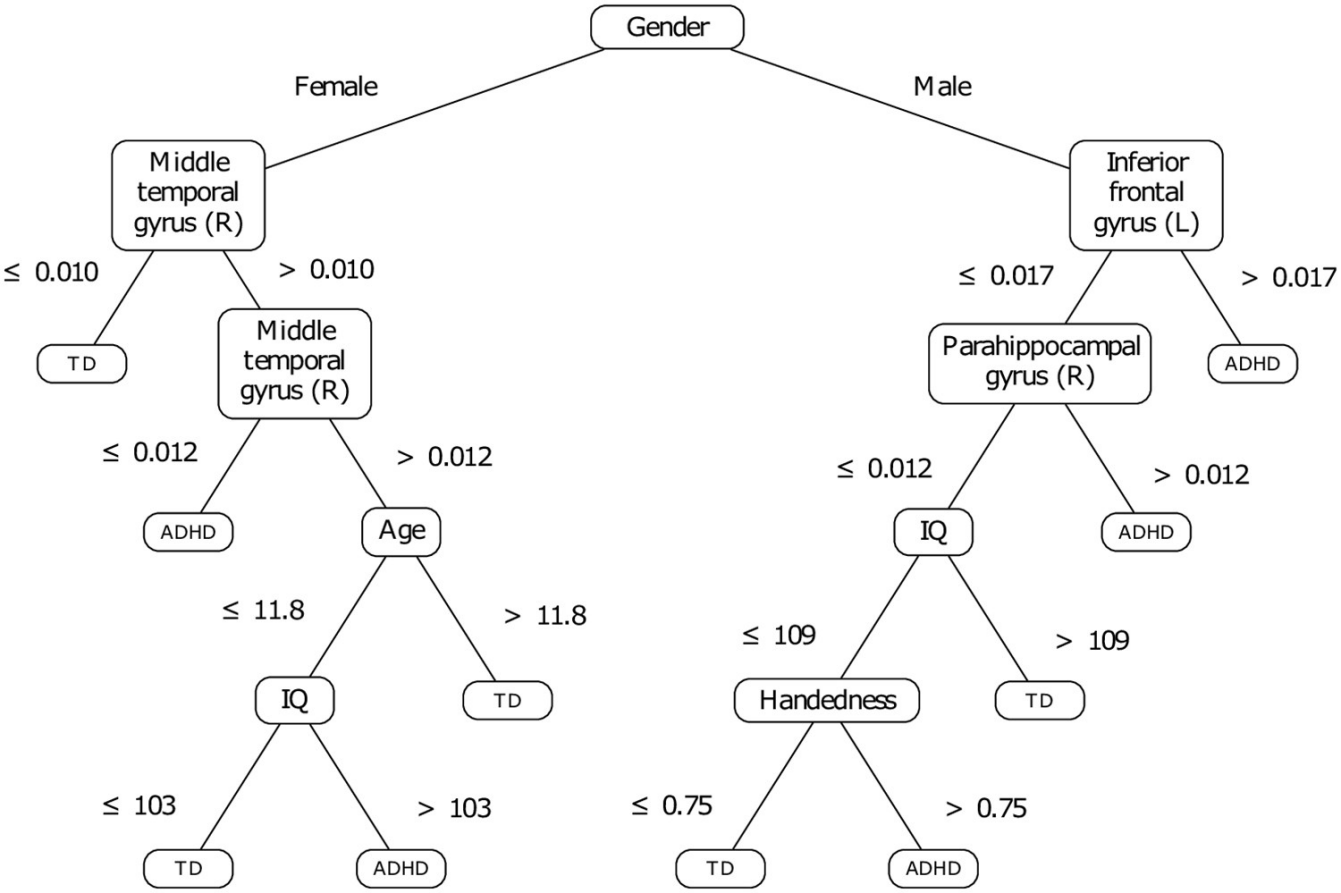
Decision tree developed without prior knowledge: Influence of the phenotypic features.

To check the relevancy of the gender among the other phenotypic features, another decision tree was trained on the whole set of phenotypic features (age, handedness, intellectual quotient and gender), as well as the same set of brain zones selected by the feature extraction process applied previously (see Table 3). The associated model is presented by Fig 6. This result confirms somehow that the phenotypic features, except gender, are less significant since they are used in the last branches of the tree. The related subdivisions replace some based on brain zones in the previous model (see Fig 5). Therefore, these phenotypic features seem to have a slightly better separative power in the context of a recursive and greedy division, taking the best decision at a local level, at each iteration. Even if the accuracy of this second model is higher than the first one, it might be possible that the age, IQ and handedness act as *overfitting factors* in the model. But it remains clear that, in keeping its position at the tree root, the gender seems to be highly discriminative. In fact, the decision tree may be seen as the association of two gender-specific classifiers, which makes sense to a certain extent. Indeed, gender-specific differences in ADHD have been widely reported in the literature related to functional and structural neuroimaging studies [76–81]. Moreover, it has long been recognized that ADHD is less prevalent in girls [1]. It appears therefore important to integrate gender to the set of training features.

### Knowledge-based ML process

Except for the right middle temporal gyrus, the regions involved in our first proposed model (see Fig 5) belong to the Papez circuit [82]. The right middle temporal gyrus may act as a *proxy* for another region in this circuit. As a matter of fact, from a computational point of view, it is possible that, in the moment of the division of the node including girls, the algorithm found more than one valid splitting attribute and thus, selected the right middle temporal gyrus arbitrarily. In view of this, it may be argued that the activation of the latter zone is a reflection of the activation of one or several other brain zones. This is in consistent relationship with the reality of the neuronal functioning, e.g. it was reported that the temporal gyrus is stimulated by the projections of the hippocampus [83, 84]. Therefore, in this stage of the study, we forced the algorithm to develop a decision tree based on regions located in the limbic system, which includes the Papez circuit. As suggested in [70], the limbic system can be thought of as a set of functional subsystems. Given the nature of the disorder studied in this work, we selected regions associated with affective and executive processes (see Table 4).

**Table 4:** List of the selected brain zones selected based on prior experiment and expert knowledge.

Affective functions	Executive functions
03-04: Superior frontal gyrus, dorsolateral	31-32: Anterior cingulate and paracingulate gyri
05-06: Superior frontal gyrus, orbital part	33-34: Median cingulate and paracingulate gyri
07-08: Middle frontal gyrus	35-36: Posterior cingulate gyrus
09-10: Superior frontal gyrus, medial orbital	37-38: Hyppocampus
13-14: Inferior frontal gyrus, triangular part	39-40: Parahippocampal gyrus
15-16: Inferior frontal gyrus, orbital part	41-42: Amygdala
	77-78: Thalamus

**Fig 7:**
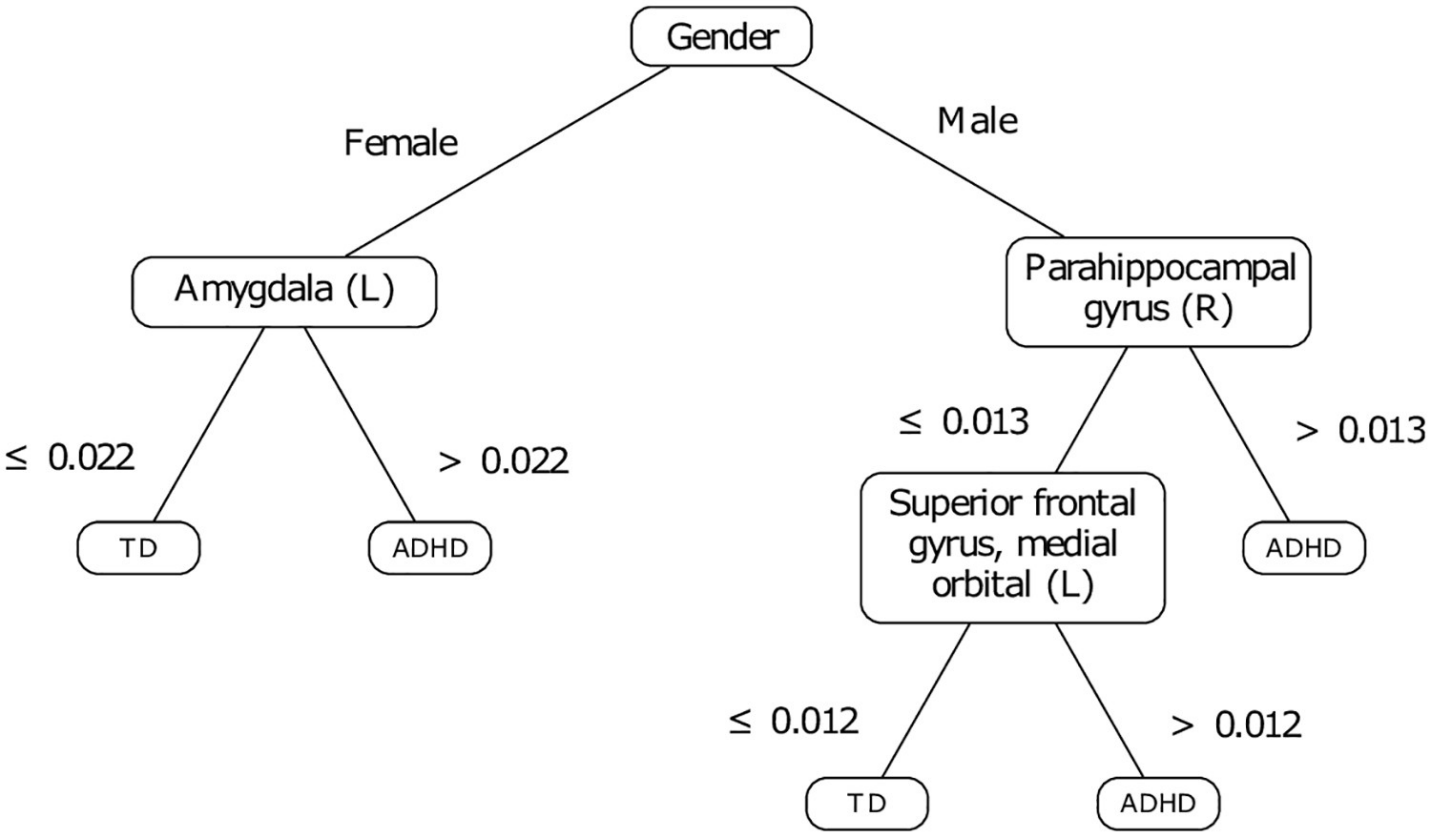
Decision tree developed based on prior knowledge.

The training set of features was thus constituted of the gender and 26 ROI. The resulting decision tree (see Fig 7) presents a 4-fold CV accuracy of 66.6±2.4%, a prediction accuracy of 73.2% on the test set (*tn* = 58.3%, *tp* = 79.3%), and is interestingly shorter. This decision tree is based on the assessment of three cerebral regions: the left amygdala in girls, the right parahippocampal gyrus and the left superior frontal gyrus, medial orbital in boys. The model supports previous findings suggesting that the amygdala plays an important role in the systemic brain pathophysiology of ADHD. For instance, [85] evidences bilaterally smaller amygdala volumes in patients with ADHD as compared to healthy controls notably.

The model keeps a discussion based on the parahippocampal gyrus for boys, the latter which was already identified in our first proposition of model (see Fig 5). Finally, boys are assessed against the left superior frontal gyrus, medial orbital. Yet it was reported that extensive damage of the orbitofrontal regions involving parts of the anterior cingulate cortex leads to impulse control problems and antisocial behaviors [86]. Thus, we can emit the hypothesis that ADHD in boys may be characterized by difficulties in the control of impulses and social conventions.

### Summary

The analysis of the first decision tree (see Fig 5) showed the involvement of brain areas previously investigated in the literature as associated with functional and/or structural irregularities with respect to ADHD. The majority of these areas are located in the limbic system. Given this first result, we constituted a training set including the gender information and the areas located in the affective and executive limbic sub-systems, as suggested by the domain expert.

The implementation of decision trees is sensitive to the training set content. While it may appear to be an inconvenient predictive model [55], this sensitivity is important when inferring a first level of knowledge, used in a second step to adjust the training set and potentially to implement a more consistent predictive model. In this case, the adjustment resulted in a decision tree which makes more sense since it does not include ternary splits which may be hardly interpreted. Admittedly, objections could be raised regarding the simplicity of the resulting decision tree. Our final proposition involves discussions on three brain regions (see Fig 7), with a prediction rate of 73.2% on the test set; Table 5 presents the related confusion matrix. This result suggests that we can not exclude the hypothesis that the neural correlates of ADHD may be explained simply. Incidentally, a model such as SVM, which is assumed to model complex conditions provides similar, and sometimes lower predictive performances if referring to previous results [26, 87, 88]. Table 6 provides a comparison with the literature on the NYU test set. The comparison reinforces our belief that our approach is interesting. Despite the unbalance in the representation of ADHD and TD subjects between both the training and the test sets, the model predicts relatively well ADHD subjects. That being said, this same unbalance has an influence in the way in which the errors are distributed between false positives and false negatives. In this regard, one interesting perspective is to find a way of adjusting the distribution of such errors. Such an approach may depend on the sensitivities of the clinician and on the pathology to be diagnosed.

**Table 5:** Confusion matrix of our predictive model on the test set.

Predicted as ⊳	TD	ADHD
TD	7	5
ADHD	6	23

**Table 6:** Comparison with previous works.

	*A*(%)	*tn*(%)	*tp*(%)
Our work	73.2	58.3	79.3
Eslami and Saed (2018) [89]	53.0	83.0	55.0
Riaz et al. (2016) [87]	61.0	41.6	68.9
Guo et al. (2014) [88]	63.8	-	-
Colby et al. (2012) [26]	37.0	58.0	34.0

## Conclusion

In the sphere of translational neuroscience, studies based on machine learning approaches have been increasing over the last years. However, few of these studies have had a clinical impact as they have still not resulted in models that aid the diagnosis of disorders such as ADHD, whose physiological bases remain unknown.

This study is on a machine learning methodology that can lead towards more interpretable models. Indeed, interpretability is an important requirement that diagnosis aid models should comply with. Decision trees are models suitable for such a purpose. Though decision trees are readable, the related decision chains do not necessarily make sense for their final user. In that regard, we showed the significance of an expert-based framework for the development of interpretable predictive models. We applied this framework on a data sample extracted from the open ADHD-200 collection. This approach is applied in two stages. An initial predictive model is developed and then analyzed by a domain expert. This allows to raise some ideas that could be explored, and to adjust, as a second stage, the training set content. Another predictive model is thus developed: it is expected to be more interpretable. Through the interaction of a domain expert, this two-stage approach likely enables to get better confidence and meaningfulness regarding the credibility of the predictive models. Let us note that we used decision trees to illustrate this approach, but the latter could be based on any classifier, as long as this classifier may be interpreted and provides enough flexibility to allow stepwise improvements of its predictive mechanism.

The first part of our study revealed a possible involvement of the Papez circuit as an element of ADHD diagnosis. Then, we investigated the credibility of this finding, based on the development of predictive models only on the areas of the limbic system, which includes the Papez circuit. The resulting decision tree presents a test accuracy of 73.2%, which constitutes a pertinent evolution with respect to the recent figures reported in the literature. Of course, the clinical significance of our results has yet to be investigated in the sphere of neuroscientific research.
